# Scenario-based estimation of economic losses associated with bovine viral diarrhea virus infection in Hanwoo and dairy cattle in Korea

**DOI:** 10.3389/fvets.2026.1973189

**Published:** 2026-10-29

**Authors:** Soo-Hee Moon, Kyoung-Seong Choi, Chang-Gi Jeong, Jae-Ku Oem

**Affiliations:** 1 Division of Agricultural Economics and Food Marketing, College of Agriculture & Life Sciences, Jeonbuk National University, Jeonju, Republic of Korea; 2 Department of Animal Science and Biotechnology, College of Ecology and Environmental Science, Kyungpook National University, Sangju, Republic of Korea; 3 Laboratory of Veterinary Infectious Disease, College of Veterinary Medicine, Jeonbuk National University, Iksan, Republic of Korea; 4 College of Veterinary Medicine, Jeonbuk National University, Iksan, Jeonbuk, Republic of Korea

**Keywords:** bovine viral diarrhea virus, dairy cattle, economic loss, Hanwoo, Monte Carlo simulation, probabilistic sensitivity analysis, veterinary economics

## Abstract

Bovine viral diarrhea virus (BVDV) can cause substantial losses in cattle production through reproductive failure, calf mortality, reduced productivity, premature culling, and additional disease-management expenditures. This study estimated the annual farm-level economic losses associated with BVDV-related production impacts under illustrative Hanwoo (Korean native beef cattle) and dairy farm scenarios in the Republic of Korea while explicitly accounting for uncertainty in key epidemiological and production-loss parameters. A probabilistic partial-budget model combined literature-derived epidemiological and production-impact parameters with Korean livestock-industry data. To reduce double counting, each loss component was applied to an event-specific population at risk. Triangular or lognormal probability distributions were assigned to uncertain inputs, and 10,000 Monte Carlo iterations were performed for each production system. Outcomes were summarized using the mean and 90% uncertainty interval (UI; P05–P95), and Spearman rank correlation coefficients were used for sensitivity analysis. For a 50-head Hanwoo farm, the mean annual scenario-based loss was estimated at KRW 12.906 million (90% UI: KRW 8.782–18.056 million), equivalent to KRW 258,113 per head (90% UI: KRW 175,646–361,121). For a 100-head dairy farm, the corresponding estimates were KRW 11.102 million per year (90% UI: KRW 7.126–16.211 million) and KRW 111,023 per head (90% UI: KRW 71,261–162,119). Sensitivity analysis showed that calf value, abortion, and calf mortality were among the model inputs most strongly associated with simulated total losses in both systems, and these factors were also among the largest contributors to economic loss. These estimates represent modeled scenarios based on literature-informed assumptions, providing a quantitative baseline rather than direct causal measurements. The model provides a quantitative baseline for future evaluation of BVDV surveillance, early detection and removal of persistently infected cattle, vaccination, biosecurity, and movement-control strategies in Korea.

## 1 Introduction

Bovine viral diarrhea virus (BVDV) is a globally distributed cattle pathogen and an economically important cause of disease in both dairy and beef production systems (1). Infection can cause enteric and respiratory disease, reproductive disorders, fetal damage, immunosuppression, and reduced productivity. At the farm level, BVDV may affect income through reduced milk yield, impaired reproductive performance, calf mortality, and increased treatment and control costs (2, 3). Infection during early pregnancy can result in persistently infected (PI) animals, which shed virus throughout life and serve as a major source for the maintenance and transmission of BVDV within herds (4, 5). Global studies have consistently shown substantial heterogeneity in the occurrence and economic effects of BVDV. A systematic review and meta-analysis of studies published between 2010 and 2021 estimated a pooled antigen prevalence of 15.74% (95% CI: 11.35–20.68) and a pooled antibody prevalence of 42.77% (95% CI: 37.01–48.63), with variation by region, breed, diagnostic method, sample type, and production system (6). Economic outcomes likewise vary according to country, production system, infection status, control policy, evaluation period, and the loss components included. Commonly reported losses include mortality, reduced conception, abortion and stillbirth, reduced milk yield, premature culling, reduced weight gain, and veterinary and control costs (1, 5, 7). One worldwide review reported annual direct monetary losses ranging from USD 0.50 to 687.80 per animal (8), while another systematic review showed that BVDV mitigation studies have evaluated control and eradication programs, monitoring and surveillance, prevention, vaccination, culling, and testing strategies (3).

Several countries have implemented BVDV control programs incorporating identification and removal of PI animals, routine testing, movement restrictions, farmer education, vaccination, and biosecurity (9–12). European eradication programs have used staged diagnostic strategies for initial herd classification, follow-up testing of positive herds, and continued monitoring of BVDV-free status (13). In Germany, after implementation of a nationwide compulsory control program, the proportion of farms with PI cattle declined from 3.44% in 2011 to 0.16% in 2016, while individual-level PI prevalence declined from 0.48 to 0.02% over the same period (10). Across such programs, early detection and removal of PI animals and prevention of infection in pregnant cattle are central principles for interrupting the generation of new PI calves (14–16).

BVDV infection has also been repeatedly reported in Korea. In a study of 319 samples from calves with clinical diarrhea in Gyeongnam Province, BVDV was detected in 12.9% of samples (17). Another study reported a BVDV antibody-positive rate of 72.2% among 4,260 Hanwoo cattle and identified 27 PI animals, 40.7% of which were calves younger than 6 months (18). A review of 81 Korean studies on infectious-disease agents in cattle published from 2010 to 2019 found a BVDV detection rate of 14.8% in calves and growing cattle, 10.3 percentage points higher than the 4.5% rate in adult cattle (19). These Korean studies reported different epidemiological measures, including virus detection, antibody positivity, and PI-animal identification. These measures represent distinct aspects of BVDV epidemiology and should not be interpreted interchangeably. Therefore, the prevalence parameter used in the present economic model was treated as a literature-informed scenario input rather than as a direct estimate of national seroprevalence or PI prevalence in Korea. These studies demonstrate the epidemiological presence of BVDV in Korea but do not provide an integrated estimate of its economic consequences under domestic production conditions. Quantitative economic assessment of BVDV in Korean Hanwoo and dairy production systems remains limited, particularly with respect to uncertainty in infection prevalence, abortion, calf mortality, milk-yield reduction, growth delay, body-weight loss, and premature culling. Therefore, the objectives of this study were to (i) estimate annual farm-level economic losses in Korean Hanwoo and dairy farms under explicitly defined, literature-informed BVDV production-loss assumptions; (ii) quantify uncertainty in those estimates using Monte Carlo simulation; and (iii) identify the input parameters most strongly associated with variation in simulated total losses. The study was not designed to evaluate the cost–benefit of a specific BVDV control program, but rather to provide a baseline for future assessment of control strategies.

## 2 Materials and methods

### 2.1 Study design and data sources

This study was designed as a probabilistic economic assessment of annual losses associated with BVDV infection in Hanwoo and dairy production systems. The BVDV prevalence parameter used in this model was defined as a scenario-based individual-level infection prevalence representing the proportion of animals assumed to be affected by BVDV for economic-loss calculations. It was not intended to represent BVDV antibody seroprevalence or the prevalence of persistently infected (PI) cattle. Biological and epidemiological inputs were derived from domestic and international studies reporting abortion, calf mortality, body-weight and milk-yield reductions, reproductive effects, and premature culling. Economic inputs, including calf prices, raw milk prices, carcass prices, replacement-animal prices, feed costs, and national cattle-industry statistics, were derived from Korean official statistics and industry data (20–23).

Illustrative herd-size scenarios were specified as 50 head for the Hanwoo model and 100 head for the dairy model. The dairy scenario assumed 80 lactating cows. These herd sizes were used as standardized modeling scenarios and were not intended to represent exact national-average herd structures (24, 25). Economic outcomes were classified as direct production losses and additional disease-related expenditures. Direct losses included abortion, calf mortality, milk-yield reduction, body-weight loss or growth delay, and net replacement costs attributable to premature culling. Disease-management and control expenditures included diagnostic testing, veterinary services, medication, labor, and herd-management costs.

Losses were estimated using an incremental partial-budget framework comparing the economic consequences under the BVDV-affected scenario with the outcomes expected under an uninfected state. Uncertainty in BVDV prevalence, abortion, calf mortality, body-weight and milk-yield reduction, duration of production losses, and premature culling was represented using probability distributions; inputs for which suitable distributional information was not available were treated as fixed values. For each production system, 10,000 Monte Carlo iterations were conducted to generate distributions of annual losses per farm and per animal.

Monte Carlo simulation was implemented in Microsoft Excel (version 2,408). Random values were generated from the specified input distributions using Excel’s built-in RAND() function. The initially generated random draws were then fixed as values and retained for subsequent calculations. Although a fixed random-number seed was not specified—meaning exact numerical point estimates may vary slightly across individual runs—the large sample size of 10,000 iterations was chosen to minimize Monte Carlo sampling error and ensure stable estimates for the mean and percentile-based uncertainty intervals. In this study, the conceptual framework of the economic-loss model, progressing from epidemiological and production-impact inputs through event-specific populations at risk, economic-loss estimation, Monte Carlo simulation, uncertainty estimation, and sensitivity analysis, is presented in Figure 1.

**Figure 1:**
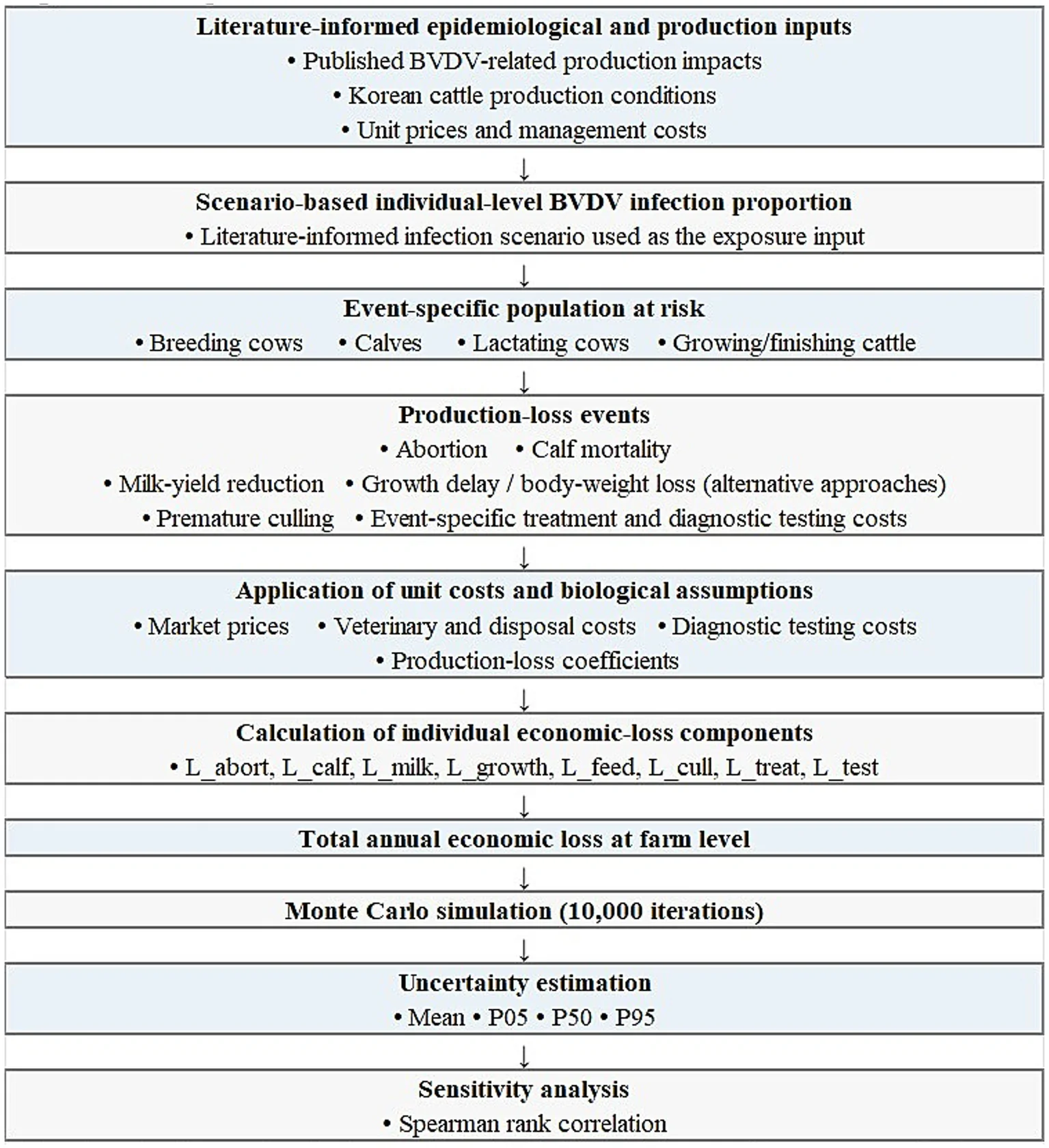
Conceptual framework of the economic-loss model. Literature-informed epidemiological and production-impact parameters were combined with a scenario-based individual-level BVDV infection proportion to identify event-specific populations at risk. Production-loss events and corresponding unit costs were then used to estimate individual economic-loss components and total annual farm-level losses. Uncertainty in key input parameters was propagated through 10,000-iteration Monte Carlo simulation, and the resulting distributions were summarized using the mean and percentile-based uncertainty estimates. Sensitivity analysis was performed using Spearman rank correlation to identify the parameters most strongly associated with variation in total simulated losses.

### 2.2 Economic loss components

BVDV-related economic outcomes were divided into production losses and additional disease-related expenditures. Production losses represented reductions in biological output or productivity attributable to the modeled BVDV effects, whereas additional disease-related expenditures represented incremental resources devoted to feed, treatment, veterinary care, labor, herd management, and diagnostic testing (7, 26–28).

Each loss component was applied to the relevant event-specific population at risk rather than to the entire herd. Abortion and postnatal calf mortality arising from the same pregnancy were treated as mutually exclusive events to avoid double counting. The abortion and calf-mortality parameters represent conditional excess probabilities attributable to the modeled BVDV effect among BVDV-infected animals, rather than the absolute probabilities of abortion or calf death from all causes. Thus, background abortion and mortality probabilities were not added again to these BVDV-attributable loss components. The population at risk for reproductive losses was defined as pregnant breeding cows, whereas calf mortality was evaluated only among live-born calves from pregnancies that did not end in abortion. Milk-yield losses were applied only to infected dairy cows that survived and remained in milk. Growth-delay losses were evaluated in infected growing cattle on the basis of reduced average daily gain (ADG), and additional feed costs were estimated as the cost required to compensate for delayed growth to the expected market weight. Veterinary, treatment, herd-management, and diagnostic-testing costs were estimated separately so that they were not repeatedly added to individual production-loss components. Definitions and model equations for each component are presented in Table 1 and Table 2.

**Table 1:** Components and equations used to estimate economic losses associated with BVDV infection.

Category	Economic loss component	Symbol	Model equation
Production loss	Abortion	L_abort	No. of breeding cows x BVDV prevalence in breeding cows × excess abortion probability among BVDV-infected breeding cows × calf price
	Calf mortality	L_calf	No. of calves at risk × BVDV prevalence in calves × excess mortality probability among BVDV-infected calves × calf price
	Milk-yield reduction	L_milk	No. of lactating cows × BVDV infection rate × (milk-yield reduction rate × duration of reduction × daily milk yield) × raw milk price
	Growth delay (reduced weight gain)	L_growth	No. of BVDV-infected growing cattle × ADG reduction × duration of reduction × carcass price (KRW/kg)
	Additional feed cost related to growth delay	L_feed	No. of BVDV-infected cattle × body-weight loss (kg/head) × feed cost per kg of body-weight gain
	Premature culling (net replacement cost)	L_cull	No. of cattle at risk × incremental premature culling rate × (replacement-animal purchase price–cull-animal salvage value)
Additional disease-related expenditure	Disposal, Veterinary, labor, and herd-management cost	L_treat	No. of animals experiencing each BVDV-attributable abortion, calf mortality or other applicable production-loss event × event-specific veterinary, medication, labor, and herd-management cost (KRW/head)
	Incremental BVDV diagnostic testing cost	L_test	No. of animals requiring diagnostic testing for each BVDV-attributable abortion, calf mortality or other applicable production-loss event × unit diagnostic-testing cost (KRW/head)

Each component was applied to its event-specific population at risk. Abortion and calf mortality from the same pregnancy were treated as mutually exclusive events. Milk-yield loss was applied only to lactating dairy cows. Veterinary, treatment, herd-management, and diagnostic-testing costs were estimated separately to avoid repeated addition to production-loss components. Accordingly, $L_{\text{treat}}$ and $L_{\text{test}}$ were not calculated simply as herd size multiplied by the BVDV infection proportion. Instead, these costs were applied only after the corresponding BVDV-attributable production-loss events had been calculated, using the number of animals experiencing each event as the relevant cost-bearing population. Treatment and testing costs were applied only to animals experiencing BVDV-attributable production-loss events, such as abortion and calf mortality, using a disposal/management cost of KRW 50,000/head, a veterinary cost of KRW 30,000/head, and a diagnostic-testing cost of KRW 20,000/head.

**Table 2:** Literature basis and rationale for stochastic input distributions used in the BVDV economic-loss model.

Parameter	Epidemiological interpretation	Literature review findings	Model application	Distribution	Reference/data source
BVDV infection proportion	Scenario-based individual-level BVDV infection proportion	Korean studies reported heterogeneous BVDV detection levels, including approximately 4.5% in adults, values around 9.6% in some datasets, and 14.8% in calves/growing cattle. Antibody positivity and PI prevalence were treated as distinct epidemiological measures and were not interpreted as equivalent to the model parameter.	5/10/15%	Triangular (min/mode/max)	(6, 17, 19)
Abortion	Excess abortion probability among BVDV-infected pregnant cows	Studies in high-producing dairy cattle reported an increase in abortion risk of about 2.03 percentage points, while other field studies reported approximately 5% additional abortion after BVDV introduction. Seroconversion-related studies reported fetal-loss estimates around 9.7%, supporting a conservative upper scenario.	2/5/10%	Triangular (min/mode/max)	(1, 26, 27, 39)
	Calf value	For Hanwoo, a baseline price of KRW 2.5 million per head was applied based on the farm-gate price of 6 to 7-month-old female calves in January 2025. For dairy cattle, KRW 0.8 million per head was applied based on the average farm-gate price of weaned male calves in normal years in South Korea.	Hanwoo: mean = 2,500 thousand KRW/head, CV = 20%, SD = 500 thousand KRW; Dairy: mean = 800 thousand KRW/head, CV = 25%, SD = 200 thousand KRW	Lognormal; Hanwoo: μ = 7.8044, σ = 0.1980; Dairy: μ = 6.6543, σ = 0.2462	(23–25)
Calf mortality	Excess mortality probability among BVDV-infected calves	An increase in calf mortality of approximately 5% after BVDV introduction has been reported, with published ranges of roughly 2–8%. Field comparisons have also reported additional mortality of approximately 5–7%.	2/5/10%	Triangular (min/mode/max)	(1, 8, 27, 40)
Weight loss	Reduction in average daily gain (ADG)	PI-BVDV exposure studies reported reductions in average daily gain (ADG) of approximately 0.15–0.22 kg/day, while stronger experimental challenge conditions reported reductions approaching 0.5 kg/day.	0.2/0.35/0.5 kg/day	Triangular (min/mode/max)	(7, 27)
Growth delay	Acute-infection-related body-weight loss rate (feed efficiency)	PI calves have been reported to show poorer growth performance than uninfected calves, and European data reported approximately 4–5% lower growth/body-weight performance during the 30-180-day growth period.	2/5/10%	Triangular (min/mode/max)	(26)
Milk yield	Milk-yield reduction rate	Some studies reported milk-yield reductions of about 5.8% in BVDV-exposed herds, while reductions greater than 10% were reported after seroconversion in other studies. More recent Dutch herd data reported smaller average reductions.	5/10/20%	Triangular (min/mode/max)	(8, 28, 39)
Duration of milk-yield loss	Duration of milk-yield reduction	Milk-yield reduction has been observed for at least approximately 10 days after seroconversion, while PI- or clinically associated production effects have been reported over roughly 1–2 weeks and, in some reports, up to 2–3 weeks.	5/10/20 days	Triangular (min/mode/max)	(28, 39)
Culling	Premature culling rate	Bio-economic studies have used heterogeneous BVDV-related premature-culling risks, including values around 2% for endemic-herd settings, up to approximately 8% for epidemic settings, and around 1.8% for mixed scenarios.	0.5/1.0/ 2.0%	Triangular (min/mode/max)	(7, 8, 26)

The minimum, most-likely, and maximum values of triangular distributions are literature-informed scenario anchors, not empirical quantiles estimated from a single homogeneous population. The modeled BVDV prevalence represents a scenario-based individual-level infection parameter and should not be interpreted as antibody seroprevalence or PI prevalence. The 5%, 10%, and 15% values were used as minimum, most-likely, and maximum scenario assumptions, respectively. For Hanwoo and dairy cattle, calf values represent baseline economic input parameters used in the scenario-based economic model, rather than annual average market prices. Calf value was modeled using production-system-specific lognormal distributions (Hanwoo: mean KRW 2.5 million/head, CV 20%; Dairy: mean KRW 0.8 million/head, CV 25%). Parameters μ and σ are reported on the natural-log scale. Fixed inputs included replacement-animal purchase price of KRW 3,000 thousand/head, cull-animal salvage value of KRW 1,500 thousand/head, carcass price of KRW 7,000/kg, feed cost for body-weight gain of KRW 3,500/kg, Hanwoo ADG of 0.8 kg/day, daily milk yield of 28 kg/head/day, and raw milk price of KRW 1,200/kg (20–23).

### 2.3 Monte Carlo simulation

Monte Carlo simulation was used to propagate uncertainty in the BVDV economic-loss model. This approach repeatedly samples values from prespecified probability distributions for uncertain input parameters and recalculates model outcomes, thereby generating a distribution of plausible economic losses rather than a single deterministic estimate (29–31). The modeled production-loss components were abortion, calf mortality, milk-yield reduction, growth delay, additional feed cost and premature culling. Veterinary treatment, medication, labor, diagnostic testing, and herd-management costs were included as additional disease-related and control expenditures (1, 7, 32). The annual total economic loss attributable to the modeled BVDV-related impacts was calculated using Equation 1:


$$\begin{array}{c} L_{\text{total}}=L_{\text{abort}}+L_{\text{calf}}+L_{\text{milk}}+L_{\text{growth}}\\ +L_{\text{feed}}+L_{\text{cull}}+L_{\text{treat}}+L_{\text{test}} \end{array}$$ (1)

where $L_{\text{total}}$ is the annual total economic loss; $L_{\text{abort}}$ is the abortion loss; $L_{\text{calf}}$ is the calf-mortality loss; $L_{\text{milk}}$ is the milk-yield reduction loss; $L_{\text{growth}}\;$ is the loss from reduced weight gain; $L_{\text{feed}}$ is the additional feed cost associated with growth delay; $L_{\text{cull}}$ is the net replacement cost associated with premature culling; $L_{\text{treat}}$ represents event-specific fixed management and veterinary costs applied only to animals experiencing abortion, calf mortality, or weight-loss events, including disposal/handling costs and veterinary service costs; and $L_{\text{test}}$ represents event-specific diagnostic testing costs applied to the same affected animals. The $L_{\text{growth}}$ and $L_{\text{feed}}$ components were not included simultaneously in the same total economic-loss calculation.

For a generic loss component i , the basic calculation is given by Equation 2:


$$L_{i}=N_{i}\times p_{\text{BVDV},i}\times E_{i}\times C_{i}$$ (2)

where $N_{i}$ is the event-specific population at risk for loss component i ; $p_{\text{BVDV},i}$ is the scenario-based individual-level BVDV infection prevalence assumed for the relevant population at risk; $E_{i}$ is the BVDV-associated conditional excess-event probability or magnitude of production impact (e.g., abortion, mortality, or culling); and $C_{i}$ is the corresponding unit economic value (e.g., calf price, raw milk price, replacement cost, or treatment cost). When a production impact was expressed as a duration or quantity rather than an event probability, a component-specific equation was used. Component-specific adjustments and additional costs are shown in Table 1.

For each Monte Carlo iteration, the number of BVDV-infected animals at risk was first calculated from the relevant risk population and the sampled BVDV prevalence. BVDV-attributable abortion and calf-mortality events were then calculated by multiplying the infected population at risk by the corresponding conditional excess-event probability. This structure prevented event probabilities from being applied to uninfected animals and restricted each event probability to the appropriate infected population at risk.

In this model, BVDV prevalence was defined as a scenario-based individual-level infection parameter representing the proportion of animals assumed to be affected by BVDV within the relevant population at risk. This parameter was used as a multiplier for BVDV-associated conditional excess production impacts. It should not be interpreted as antibody seroprevalence or as the prevalence of persistently infected animals. Because nationally representative Korean estimates corresponding precisely to this model parameter were unavailable, a triangular distribution of 5%, 10%, and 15% was used as a literature-informed scenario range. Variables with substantial biological uncertainty were modeled using triangular distributions parameterized by minimum, most likely, and maximum values. Triangular distributions were used for uncertain biological and production-impact parameters for which the available literature provided plausible lower, central, and upper values or ranges but insufficient information to estimate a parametric sampling distribution directly. Accordingly, the minimum, mode, and maximum values should be interpreted as literature-informed scenario anchors rather than empirical quantiles or parameters estimated from a single homogeneous population. These included excess abortion probability, excess calf-mortality probability, ADG reduction, body-weight loss, milk-yield reduction, duration of milk-yield loss, and premature culling.

Calf price was modeled separately for Hanwoo and dairy cattle using lognormal distributions because market prices are strictly positive and may exhibit right-skewed variability. Replacement-animal purchase price, cull-cow salvage value, feed cost, baseline ADG, diagnostic and management costs, daily milk yield, and raw milk price were treated as fixed values on the basis of previous studies and Korean livestock statistics. The stochastic inputs and distributional assumptions used to estimate BVDV-related economic losses are summarized in Figure 2.

**Figure 2:**
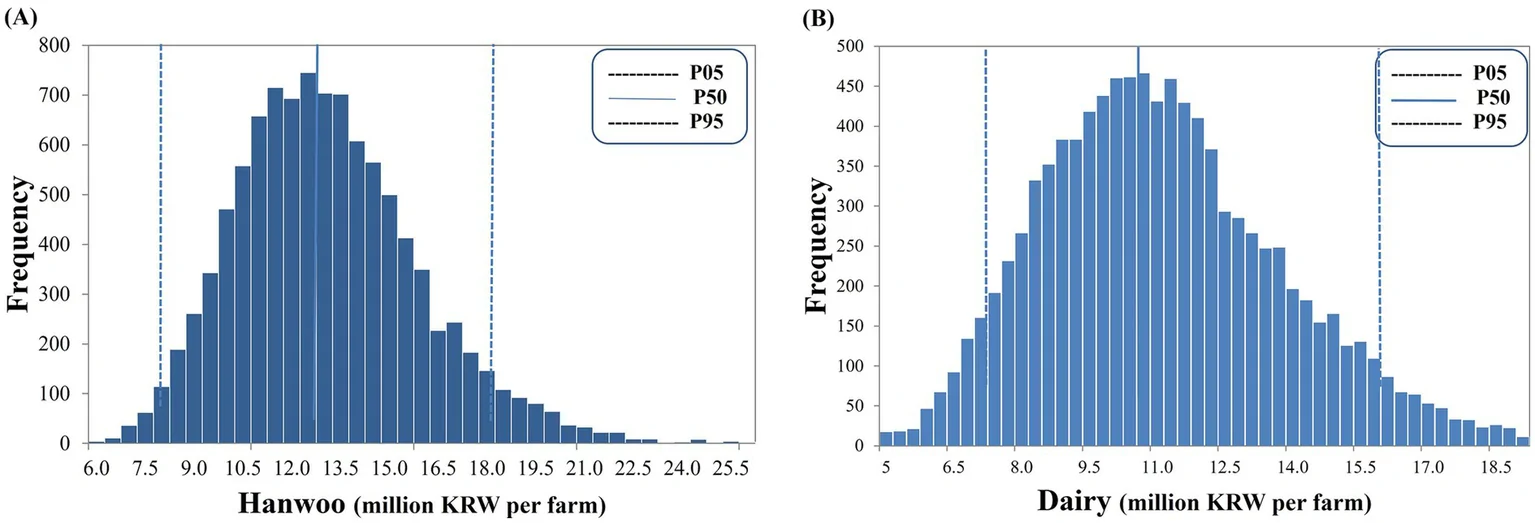
Distributions of simulated annual economic loss per farm in **(A)** Hanwoo and **(B)** dairy production systems. Vertical reference lines indicate the 5th percentile (P05), median (P50), and 95th percentile (P95) of the simulated loss distribution.

The model was run for 10,000 iterations for each production system. The resulting total-loss distributions were summarized by the mean, median (P50), 5th percentile (P05), and 95th percentile (P95). P05 and P95 were reported as the lower and upper limits, respectively, of the 90% uncertainty interval (UI). Compared with deterministic analysis based on a single point estimate, this probabilistic approach explicitly incorporates parameter uncertainty and provides a distribution of plausible losses; such methods have been used in economic evaluations of livestock diseases (33, 34).

### 2.4 Sensitivity analysis

Sensitivity analysis was conducted to identify the production-impact inputs most strongly associated with uncertainty in estimated economic losses. Because calf price was treated as a stochastic economic input, the sampled calf-price values were also included in the Spearman rank-correlation sensitivity analysis. Separate production-system-specific calf-price distributions were used for Hanwoo and dairy cattle. Spearman rank correlation coefficients were calculated between each sampled input variable and the corresponding simulated total economic loss across Monte Carlo iterations to assess the relative influence of each input (35–38).

A larger absolute sensitivity coefficient indicates a stronger monotonic association between the input variable and estimated total economic loss, while the sign indicates the direction of the association. Because rank correlation is not a variance-decomposition method, these coefficients were interpreted as measures of association rather than as proportions of total outcome variance explained. The sensitivity coefficient was calculated using Equation 3:


$$S_{i}=\rho_{s}\;(X_{i},L_{\text{total}})$$ (3)

where $S_{i}$ is the sensitivity coefficient for input i ; $X_{i}$ is the sampled value of the input variable (e.g., calf value, excess abortion probability, premature culling rate, or body-weight loss); $L_{\text{total}}$ is the simulated total economic loss; and $\rho_{s}$ is the Spearman rank correlation coefficient.

## 3 Results

### 3.1 Estimated economic losses by production system

The Monte Carlo loss distributions were unimodal. The Hanwoo farm distribution showed greater dispersion and slight right skewness compared with the dairy farm distribution. Hanwoo farms exhibited a relatively higher mean loss and greater variability, whereas the dairy model produced a narrower and more stable distribution (Figure 3).

**Figure 3:**
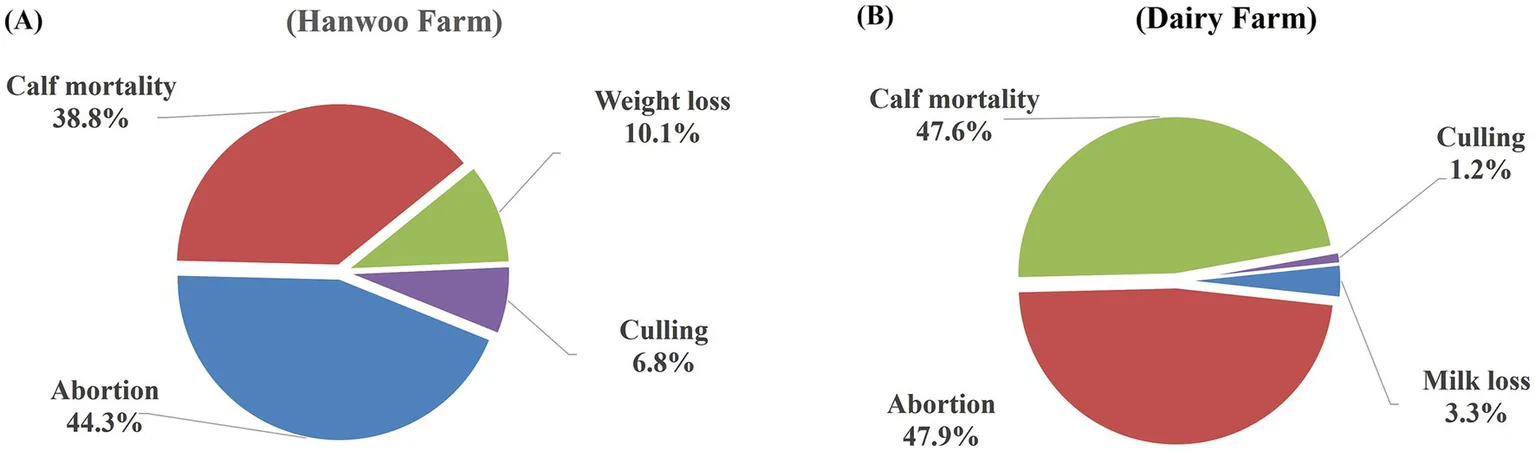
Mean-based composition of simulated annual economic losses by component in **(A)** Hanwoo and **(B)** dairy farms. The proportion for each component was calculated using the mean total economic loss for the corresponding production system as the denominator.

Under the specified BVDV-related production-loss assumptions, the mean annual scenario-based economic loss for a 50-head Hanwoo farm was estimated at KRW 12.906 million (90% UI: KRW 8.782–18.056 million), equivalent to KRW 258,113 per head (90% UI: KRW 175,646–361,121). Abortion (44.3%) and calf mortality (38.8%) together accounted for 83.1% of the modeled loss. These were followed by body-weight loss (10.1%) and premature culling (6.8%; Figure 3A and Table 3). These findings indicate that reproductive loss and calf mortality accounted for the majority of the modeled economic burden in the Hanwoo production system.

**Table 3:** Estimated economic losses by component for a Hanwoo farm in the BVDV scenario model.

Component	Percentiles of the simulated distribution		
	P05 (lower)	P50 (median)	P95 (upper)
Herd size (head)	50	50	50
Live-born calves (head)	45	45	45
BVDV infection proportion (%)	6.6	10.0	13.4
Excess abortion probability (%)	3.1	5.5	8.6
Excess calf-mortality probability (%)	3.2	5.7	8.7
Body-weight loss rate (%)	3.2	5.7	8.7
Incremental premature culling rate (%)	0.7	1.1	1.7
Calf value (KRW/head)	1.809,090	2.506,824	3,472,551
Abortion loss (KRW)	2,901,321	5,505,275	9,378,629
Calf-mortality loss (KRW)	2,996,904	4,821,737	7,448,149
Body-weight loss (KRW)	675,017	1,258,072	2,104,186
Growth-delay loss (KRW)	377,927	678,239	1,112,118
Premature-culling loss (KRW)	517,773	847,914	1,294,278
Total economic loss (body-weight loss, KRW/farm)	8,782,295	12,604,139	18,056,042
Total economic loss (growth delay, KRW/farm)	7,339,484	11,086,604	16,402,254
Total economic loss (culling, KRW/farm)	8,673,858	12,594,184	17,993,164
Total economic loss (KRW/head)	175,646	252,083	361,121
Mean economic loss (KRW/farm)	12,905,648		
Mean economic loss (KRW/head)	258,113		

Economic losses are summarized using the 5th percentile (P05), median (P50), and 95th percentile (P95) of the simulation results. Mean values represent the expected economic loss per farm and per head. Percentiles for individual components and total loss are marginal summaries of their respective distributions and should not be summed across rows. Body-weight loss and growth-delay loss represent alternative approaches to valuing growth-related losses and were not included simultaneously in the same total economic-loss estimate.

For a 100-head dairy farm, the mean annual scenario-based loss was estimated at KRW 11.102 million (90% UI: KRW 7.126–16.211 million), equivalent to KRW 111,023 per head (90% UI: KRW 71,261–162,119). Abortion and calf mortality accounted for the largest shares of loss, at 47.9 and 47.6%, respectively, followed by milk-yield loss (3.3%) and premature culling (1.2%; Figure 3B and Table 4). As in the Hanwoo model, the dairy results indicate that reproductive losses and calf mortality were the principal contributors to the modeled farm-level economic losses.

**Table 4:** Estimated economic losses by component for a dairy farm in the BVDV scenario model.

Component	Percentiles of the simulated distribution		
	P05 (lower)	P50 (median)	P95 (upper)
Herd size (head)	100	100	100
Lactating cows (head)	80	80	80
BVDV infection proportion (%)	6.5	10.0	13.5
Milk-yield reduction rate (%)	6.9	11.4	17.2
Duration of milk-yield loss (days)	6.9	11.3	17.3
Excess abortion probability (%)	3.1	5.5	8.7
Excess calf-mortality probability (%)	3.1	5.5	8.6
Incremental premature culling rate (%)	0.7	1.1	1.7
Calf value (KRW/head)	534,657	801,802	1,200,963
Abortion loss (KRW)	2,527,200	4,743,915	8,081,223
Calf-mortality loss (KRW)	2,475,737	4,713,329	8,273,384
Milk-yield loss (KRW)	156,366	336,408	671,971
Premature-culling loss (KRW)	62,333	115,499	197,389
Total economic loss (KRW/farm)	7,126,091	10,808,504	16,211,914
Total economic loss (KRW/head)	71,261	108,085	162,119
Mean economic loss (KRW/farm)	11,102,325		
Mean economic loss (KRW/head)	111,023		

Economic losses are summarized using the 5th percentile (P05), median (P50), and 95th percentile (P95) of the simulation results. Mean values represent the expected economic loss per farm and per head. Percentiles for individual components and total loss are marginal summaries of their respective distributions and should not be summed across rows.

### 3.2 Sensitivity analysis of the BVDV economic-loss model

Sensitivity analysis was performed to identify key factors associated with variation in estimated BVDV-related economic losses, including calf value, BVDV infection proportion, calf-mortality, abortion, body-weight loss, milk-yield reduction, and premature culling.

In the Hanwoo model, calf value showed the strongest association with total economic loss (Spearman correlation coefficient = 0.662), followed by excess abortion probability (0.598), excess calf-mortality probability (0.348), BVDV infection proportion (0.182), body-weight loss (0.125) and premature culling (0.079). For descriptive interpretation within this model, correlation coefficients exceeding 0.5 were considered to indicate relatively strong associations with variation in simulated total losses. Thus, excess abortion probability showed a relatively strong association with total economic loss in the Hanwoo model.

In the dairy model, calf value (0.640), excess abortion probability (0.518), and excess calf-mortality probability (0.503) were the most influential input variables, followed by BVDV infection proportion (0.062), premature culling (0.017) and milk-yield loss (0.012). These results indicate that reproductive loss and calf mortality were the production-impact inputs most strongly associated with modeled BVDV-related economic losses in both production systems (Figure 4).

**Figure 4:**
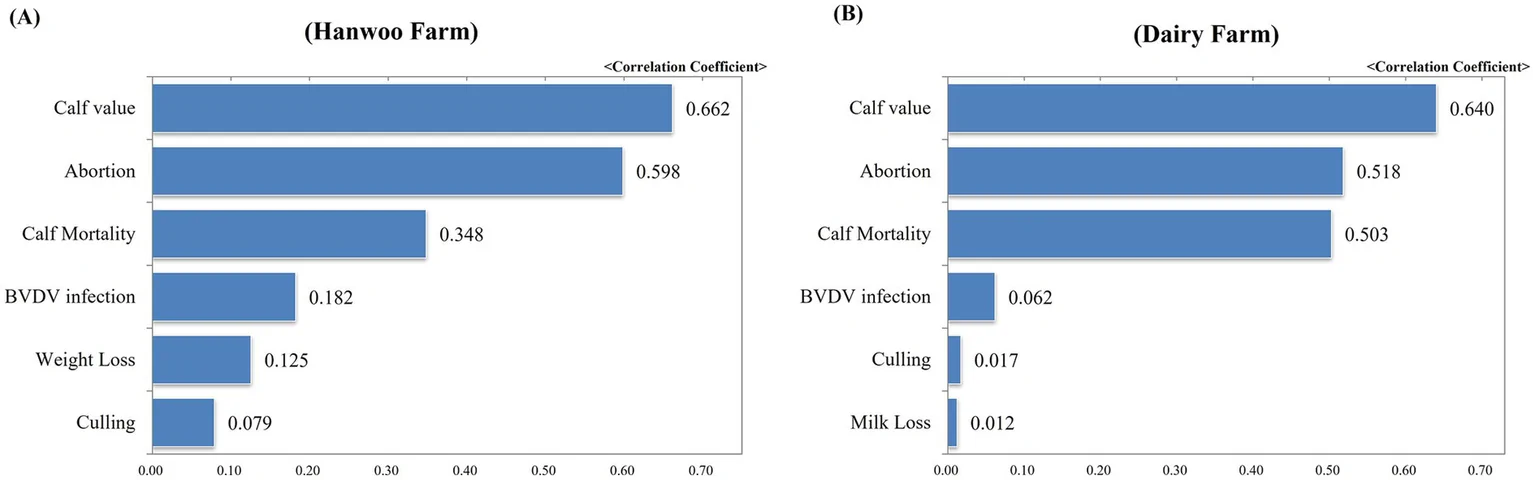
Sensitivity analysis of estimated economic losses in the BVDV scenario model. Bars represent Spearman rank correlation coefficients between the reported stochastic production-impact inputs and annual total economic loss in **(A)** Hanwoo and **(B)** dairy farms. Larger absolute coefficients indicate stronger monotonic associations with simulated total loss.

## 4 Discussion

This study used a probabilistic economic-loss model that explicitly represented uncertainty in major epidemiological and production-impact parameters to estimate annual economic losses associated with BVDV-related impacts under illustrative scenario-based Hanwoo and dairy farm scenarios in Korea. Under the specified literature-informed assumptions, the estimated annual mean loss was approximately KRW 12.91 million per Hanwoo farm and KRW 11.10 million per dairy farm, corresponding to KRW 258,113 and KRW 111,023 per head, respectively. These findings indicate that, under the modeled scenarios, BVDV-related reproductive failure, calf mortality, productivity loss, and premature culling can impose a meaningful economic burden on cattle farms.

In the Hanwoo model, abortion and calf mortality accounted for 44.3 and 38.8% of total modeled economic loss, respectively, for a combined share of 83.1%. Losses associated with body-weight reduction, premature culling, and growth delay were smaller, but remain economically relevant because they directly affect market value and the duration of production. In the dairy model, abortion and calf mortality represented 47.9 and 47.6% of total loss, respectively, together accounting for approximately 95.5%, followed by milk-yield reduction and premature culling. This pattern is consistent with previous studies reporting that the economic consequences of BVDV are driven largely by abortion, stillbirth, calf mortality, impaired reproductive performance, and reduced productivity (1, 7, 8, 26).

Direct comparison of the absolute loss estimates from this study with estimates from other countries should be made cautiously. Previous reviews have shown substantial variability in BVDV loss estimates because of differences in infection pressure, herd structure, output prices, included loss components, vaccination and control status, and evaluation period (3, 8). Accordingly, the estimates reported here are most appropriately interpreted as scenario-based values for representative Korean production systems under the model assumptions specified in this study, rather than as directly observed national causal loss estimates. An additional limitation is that stochastic input parameters were sampled independently. In biological settings, BVDV infection proportion, reproductive failure, calf mortality, and production effects may be correlated. Consequently, the reported uncertainty intervals may either understate or overstate the joint uncertainty in total economic losses, particularly in the tails of the simulated distribution. Future models should incorporate correlated sampling or joint distributions when suitable Korean epidemiological data become available.

In the sensitivity analysis, the Hanwoo model showed that calf value had the strongest association with simulated total economic loss, followed by excess abortion probability. Similarly, in the dairy model, calf value, excess abortion probability, and excess calf-mortality probability showed strong associations with total economic loss. In addition, when considering only the epidemiological and production-impact parameters, excess abortion probability showed the strongest association with total economic loss. From a research perspective, these results suggest that improved Korean field estimates of BVDV-attributable reproductive failure and calf mortality would be particularly valuable for reducing uncertainty in future economic assessments. Because rank-correlation analysis identifies monotonic associations rather than decomposing model variance, the coefficients should not be interpreted as causal effects or as proportions of variance explained.

This study has several limitations. First, long-term Korean farm-level data directly comparing BVDV-affected and unaffected herds were limited, and several epidemiological and production-impact inputs were therefore derived from domestic and international literature and represented as scenario distributions. Second, because production-group-specific prevalence estimates were unavailable, the same sampled individual-level BVDV prevalence was applied to breeding cows and calves. The estimated economic losses in our study are not a definitive figure reflecting the actual national prevalence, but rather a conditional outcome based on hypothetical infection scenarios ranging from 5 to 15%. Third, some Korean unit prices and management costs were treated as fixed values because suitable empirical distributions were unavailable. Fourth, the model used representative herd sizes and a static annual partial-budget structure and therefore did not capture within-herd transmission, repeated infection, temporal changes in prevalence, replacement dynamics, market feedback, or industry-level price effects. Fifth, the reported uncertainty intervals reflect only the uncertainty represented by the variables and distributions included in the model and do not capture all possible structural or parameter uncertainty.

Future research should collect longitudinal Korean farm data on BVDV infection, reproductive outcomes, calf survival, milk yield, growth performance, culling, use of diagnostic testing, and control expenditures. Integrating such data with dynamic BVDV transmission models would allow intervention effects to be evaluated explicitly over time. Subsequent cost-effectiveness analyses should incorporate transmission, diagnostic-test performance, vaccination or biosecurity effectiveness, PI removal strategies, program implementation costs, and production-system-specific economic outcomes.

## 5 Conclusion

Under the specified literature-informed assumptions, BVDV-related production impacts generated substantial annual scenario-based economic losses in the illustrative Hanwoo and dairy farm scenarios evaluated in this study. Abortion and calf mortality accounted for the largest shares among the modeled loss components and were also among the production-impact inputs most strongly associated with simulated total losses. Because these estimates were generated from a probabilistic scenario model rather than from direct comparisons of Korean herds, they should be interpreted as a baseline range for economic evaluation rather than as observed causal losses. Improved domestic epidemiological and production data, combined with dynamic transmission and intervention models, are needed to quantify the cost-effectiveness of BVDV surveillance and control strategies in Korea.

## Data Availability

The data used to parameterize the model were obtained from published literature and publicly available secondary sources, as described in the manuscript.The model input data and simulation outputs supporting the findings of this study are available from the corresponding author upon reasonable request.
